# The Impact of Concomitant Neck of Femur Fractures and Upper Limb Fractures on Length of Stay and Key Performance Indicators: A Single-Centre Study

**DOI:** 10.7759/cureus.60862

**Published:** 2024-05-22

**Authors:** Daniel A Lewandowski, Abdul Badurudeen, Tim Matthews

**Affiliations:** 1 Trauma and Orthopaedics, University Hospital of Wales, Cardiff, GBR

**Keywords:** average length of hospital stay, neck of femur fractures, upper limb orthopaedic surgery, key performance indicators, elderly hip fractures

## Abstract

Background

Hip fractures are one of the most common serious injuries seen today and constitute one of the most serious healthcare problems affecting the elderly worldwide. Due to the elderly population, associated falls and osteoporosis increase the incidence of hip fractures. Patients may remain hospitalized for several weeks, leading to one and a half million hospital bed days used each year. The reported incidence of a concurrent upper limb and a lower limb fracture is between 3% and 5%. It has been shown in the literature that patients who sustain both a hip fracture and an upper limb fracture have difficulties with rehabilitation which causes prolonged stays. The available literature on concomitant hip fracture and upper extremity fracture is limited. This study aimed to review patients with concurrent upper limb injury and hip fractures and to analyse the pattern of associated upper limb fractures, management of these fractures, length of hospital stay, mortality rates, and complications.

Methodology

We performed a retrospective data collection of all patients with a concomitant upper limb fracture and hip fracture from January 2017 to December 2020 at the University Hospital of Wales, Cardiff, United Kingdom. Patients were identified from the registers maintained in the ward. All patients aged over 60 years with a fragility hip fracture (managed operatively) and a concurrent upper limb fracture were included in the study. Patients aged less than 60 years were excluded. The local research department registered and approved this study as a service evaluation and therefore did not need ethical committee approval. The anatomical location of the upper limb and hip fractures was confirmed using the imaging database (Synapse).

Results

Of the 760 patients admitted with neck of femur fractures during this period, 39 (5.1%) patients had concomitant upper limb fractures. Only one upper limb fracture was managed with fixation, and for this study, that patient was excluded. Our retrospective search identified 38 patients, of whom 11 were men and 27 were women. Distal radius fractures were the most commonly associated upper limb fractures (55%). There was a significant increase in length of stay (43.6 days vs. 16.6 days) and delay in mobilization (58.9% vs. 81%) compared to an isolated hip fracture. There was no difference in the 30-day mortality rates. We were unable to collect the data for the Key Performance Indicator (KPI) of the National Institute for Health and Care Excellence compliant surgery, and this KPI was excluded from our study. Of the remaining five KPIs, our group of patients displayed better averages in three of the five categories, including prompt orthogeriatric review (92%), not delirious postoperatively (87%), and return to original residence (79%).

Conclusions

Due to the ageing population, hip fractures are increasing, and within one year of operation, have shown higher mortality rates. Annually, reports show that the worldwide incidence of fractures in the adult population ranges between 9.0 and 22.8 per 1,000. These fractures are more frequent in osteoporotic patients with weak bone quality. Following hip fractures, upper extremity fractures are the second most common among the osteoporotic, elderly population, with distal radius fractures being the most common. With the length of stay almost tripled (from 16.6 to 44.4 days), one can see this has a very big effect on costs in the National Health Service system.

## Introduction

Hip fractures are one of the most common serious injuries seen today and constitute one of the most serious healthcare problems affecting the elderly worldwide [1]. Due to higher rates of osteoporosis and falls seen in the elderly population, the incidence of hip fractures has increased [2]. Even trivial injuries such as falls from standing height can place elderly patients at risk for injuries such as hip, distal radius, proximal humerus, and spine fractures [3,4].

Patients may remain hospitalized for several weeks, leading to one and a half million hospital bed days used each year [5]. Although the overall length of stay has not significantly fallen since 2016, patients recovering from hip fractures still occupy 1 in 45 of all hospital beds in England and Northern Ireland, and 1 in 33 hospital beds in Wales [5]. It is known that elderly patients with hip fractures have high rates of morbidity and mortality as a result of which they have a significant economic impact and increasing burden on the public healthcare sector [6-8]. By 2025, the annual number of hip fractures is projected to be around 104,000 in the United Kingdom alone. Data have shown that social and health costs associated with hip fractures are enormous in the United Kingdom, ranging from £2 billion to £3 billion from previous years [2].

Prompt mobilisation after surgery is the key to patients’ successful return to baseline activities and residence. The National Hip Fracture Database (NHFD) shows that one in five patients is still not able to get out of bed on the day after surgery [5]. Although early mobilisation following a hip fracture fixation depends on a patient’s pre-injury ambulation status, associated comorbidities, frailty, and surgical and anaesthetic factors, associated upper limb fractures are examples of other limiting factors.

The reported incidence of a concurrent upper limb and a lower limb fracture is between 3% and 5% [9-13]. In the osteoporotic, elderly population, hip fractures are most common, followed by upper extremity fractures [14]. Patients who sustain both an upper limb injury and a hip fracture may have difficulty with rehabilitation and have prolonged outcomes compared to patients with just isolated hip fractures.

The available literature on concomitant hip fracture and upper extremity fracture is limited. This study aimed to review patients with concurrent upper limb injury and hip fractures and analyse the pattern of associated upper limb fractures, management of these fractures, length of hospital stay, mortality rates, and associated complications. The results are compared to the NHFD, United Kingdom. The NHFD has been publishing information on patient outcomes following hip fractures in England, Wales, and Northern Ireland since 2007 [5]. Furthermore, Key Performance Indicators (KPIs) were analysed and compared to the latest national averages. KPIs aim to describe the most important aspect of patient care, including prompt orthogeriatric review, prompt surgery, National Institute for Health and Care Excellence-compliant surgery, prompt mobilisation after surgery, not delirious when tested, and return to original residence [5].

This study aimed to investigate the prevalence of concomitant hip and upper limb fractures and how this affects the time for rehabilitation and length of stay in the hospital. It also aimed to examine how KPIs compare to the national averages supplied by the NHFD. When treating patients with concurrent upper limb injuries and hip fractures, clinicians should be aware that these patients will likely have a longer stay in the hospital than patients who have isolated hip fractures only.

## Materials and methods

We performed a retrospective data collection of all patients who had a concomitant upper limb fracture and hip fracture from January 2017 to December 2020 at the University Hospital of Wales, Cardiff, United Kingdom. Patients fulfilling the criteria were identified from the registers maintained in the ward. All patients aged over 60 years with a fragility hip fracture (managed operatively) and a concurrent upper limb fracture were included in the study. Patients aged less than 60 years were excluded from our study.

Patient demographics, type of hip fracture and upper limb fracture, management of hip and upper limb fractures, American Society of Anesthesiologists (ASA) classiﬁcation, duration of stay in the hospital, peri-operative complications, inpatient mortality, and 30-day and one-year mortality were analysed.

Patient data were obtained from the hospital’s trauma registry on patient demographics, mechanism of the injury, location of the injury, nature of the injury, and the procedure or fixation undertaken to treat the fracture. Length of stay was determined by checking the patient’s notes and the hospital’s registry to determine admission and discharge dates. The local research department registered and approved this study as a service evaluation and therefore did not need ethical committee approval.

The anatomical location of the upper limb and hip fractures was confirmed using the imaging database (Synapse). Plain film X-rays were the modality of choice to identify hip fractures, usually done in both anteroposterior and lateral views, as well as upper extremity fractures. Some patients did not have X-rays but computed tomography (CT) scans, and fracture patterns were identified and grouped based on CT scans. The operation notes of the individual patients were reviewed to confirm the nature of treatment offered to the patient (BlueSpier). Theatre records and notes were reviewed regarding the anatomical location and treatment of both hip and upper limb fractures, history of previous fractures, demographic data, the mechanism of injury, and the length of hospital stay. Patients sustaining hip fractures because of high-energy trauma, typically seen in younger age groups, were excluded. Patients who died during their acute hospital management of the concurrent upper limb fracture were also recorded in our study.

KPIs were accessed online on the NHFD for the University Hospital of Wales and compared to the national averages for that period.

## Results

**Figure 1:**
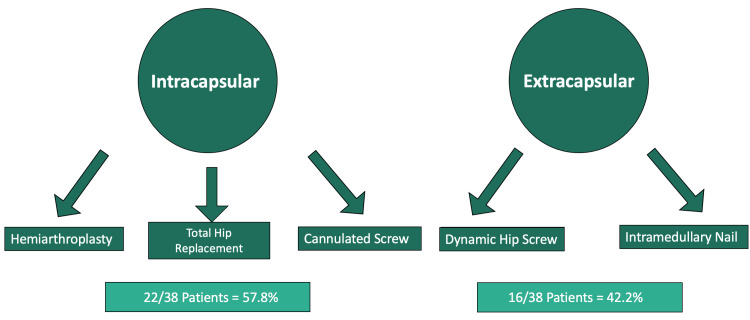
Patients divided into intracapsular vs extracapsular fractures

Of the 760 patients admitted with neck of femur fractures during this period, 39 (5.1%) patients were identified to have concomitant upper limb fractures. All 760 patients with neck of femur fractures were managed operatively. There were 16 extracapsular and 22 intracapsular fractures of the hip (see Figure 1). Only one upper limb fracture was managed with fixation, and for this study, that patient was excluded. Our retrospective search identified 38 patients, of whom 11 were men and 27 were women. The mean age of our cohort was 79.8 years (49-96 years). Overall, 37 out of 38 patients had an ipsilateral upper limb fracture, and one patient had a fracture in the contralateral upper limb.

Operations performed included dynamic hip screws, cannulated screw fixation, intramedullary nails, hemiarthroplasties, and total hip arthroplasties depending on individual factors, including fracture pattern, patient mobility, and patient age (see Table 1).

**Table 1:** Neck of femur fracture anatomical locations and associated operations performed.

Location of neck of femur fracture	Operation performed for fracture pattern	Number of patients
Intracapsular (22 total)	Hip hemiarthroplasty	16
	Total hip replacement	3
	Cannulated screws	3
Extracapsular (16 total)	Intramedullary nail	6
	Dynamic hip screw	10

The associated upper limb injuries comprised 16 proximal humerus fractures, 21 distal radius fractures, and 1 elbow fracture. Proximal humerus fractures were commonly managed conservatively with an arm sling. Patients with distal radius fractures were manipulated under the same anaesthesia and a back slab was applied. Distal radius fractures were the most commonly associated upper limb fractures (55%). There was a significant increase in length of stay (43.6 days vs. 16.6 days) and delay in mobilization (58.9% vs. 81%) in patients with upper limb fractures compared with patients presenting with an isolated hip fracture. Women had a longer length of stay compared to their male counterparts (38.2 days​ vs. 45.8 days). There was no difference in the 30-day mortality rates among patients.

KPIs were analysed in the study. At the time of our study, the NHFD used six KPIs (prompt orthogeriatric review, prompt surgery, NICE-compliant surgery, prompt mobilisation, not delirious postoperatively, and return to the original residence), but that has now expanded to eight to include admission to a specialist ward and bone medication prescribed [5]. Regarding KPIs, we compared our sample of patients (see Table 2) to the average KPIs for neck of femur fractures in the University Hospital of Wales (see Table 3), as well as to the averages provided by the NHFD (see Table 4).

**Table 2:** KPIs of patients with both neck of femur and upper limb fractures. KPI = Key Performance Indicator; NICE = National Institute for Health and Care Excellence; NHFD = National Hip Fracture Database

KPI	Patient population identified (%)	University Hospital of Wales (%)	NHFD (%)
Prompt orthogeriatric review	92	75	86
Prompt surgery	66	65	69
Prompt mobilisation	68	77	81
Not delirious postoperatively	87	60	58
Return to the original residence	79	75	71

**Table 3:** KPI overview at the University Hospital of Wales. Annualised values based on 476 cases averaged over 12 months to the end of September 2021. KPI = Key Performance Indicator; NICE = National Institute for Health and Care Excellence; NHFD = National Hip Fracture Database

KPI	University Hospital of Wales (%)	NHFD overall (%)
Prompt orthogeriatric review	75	89
Prompt surgery	65	68
NICE-compliant surgery	77	71
Prompt mobilisation	77	81
Not delirious postoperatively	60	61

**Table 4:** Average KPIs of all UK hospitals. Annualised values based on 61,835 cases averaged over 12 months to the end of February 2021. KPI = Key Performance Indicator; NICE = National Institute for Health and Care Excellence; NHFD = National Hip Fracture Database

KPI	University Hospital of Wales (%)	NHFD overall (%)
Prompt orthogeriatric review	86	86
Prompt surgery	69	69
NICE-compliant surgery	71	71
Prompt mobilisation	81	81
Not delirious postoperatively	58	58

We were unable to collect the data for the KPI of NICE-compliant surgery, and this KPI was excluded from our study. Of the remaining five KPIs, our group of patients displayed better averages in three of the five categories, including prompt orthogeriatric review (92%), not delirious postoperatively (87%), and return to the original residence (79%). Although patients scored less in prompt surgery (66% vs. NHFD 68%) and prompt mobilisation (68% vs. NHFD 81%), these results were not far off from the national averages.

## Discussion

Due to the ageing population, hip fractures are becoming more frequent and are associated with high mortality rates within one year of operation [15,16]. The worldwide incidence of fractures annually in the adult population is reported to range between 9.0 and 22.8 per 1,000 [17]. By 2050, it is estimated that the number of hip fractures may reach 6.26 million, and they are seen as the most common fractures in the elderly and frail population [18]. Patients who have osteoporosis are found to fracture more frequently than those without due to their poor bone quality [19,20]. Following hip fractures, upper extremity fractures are the second most common in the osteoporotic elderly population. Distal radius fractures are the most common upper extremity fractures. Patients with both hip and upper extremity fractures had longer lengths of stay, higher risk of in-hospital mortality, and were less likely to be discharged home when referring to their original place of residence [21].

In the literature, previous studies have evaluated the elderly population concerning outcomes and prognosis of hip fractures and upper extremity fractures. In the literature, there are limited studies on concomitant hip fractures and upper extremity fractures. One of the larger studies compiled by Morris et al. [22] examined 6,887 patients and found that 307 patients had an associated upper limb fracture (6.1%). Patients had an increased length of stay in the hospital (21.7 days vs. 18.8 days, p = 0.003). A study by Kang et al. [10] similarly showed a prevalence of 3.4% of patients with concomitant neck of femur and upper limb fractures between 2008 and 2018. Researchers found that the length of stay in the hospital was on average four days longer, with an average stay of 19.8 days. A further study by Ng et al. [9] identified 820 patients with hip fractures, 34 of whom had an upper extremity fracture (4.1%) and had significantly longer rehabilitation (34.6 vs. 19.9 days, p = 0.009). Our study follows these trends for increased length of stay in the hospital, but the duration for our patients was almost tripled (43.6 days vs. 16.6 days) compared to the other studies identified.

Some studies have examined the differences between fixation of the upper limb fractures to speed up recovery and decrease the length of stay in the hospital. While studies of surgical versus conservative treatment of distal radius fractures appeared to show equivalence in the long term, the pain suffered by the patients in their early healing phase cannot be overlooked. Although there is literature evidence available to support in favour of operative fixation of upper limb fractures in the geriatric population, limited data is available on whether it is beneficial in fixing concomitant upper limb fractures associated with a hip fracture in the geriatric population.

Saving et al. revealed that distal radius fractures when repaired surgically showed quicker recovery in the first three months postoperatively. This was due to not having prolonged plaster immobilisation and allowing the patient to mobilise their upper extremities earlier. Patients were able to return to daily living activities faster in this elderly cohort [23]. Multiple researchers have published their results on isolated distal radius fracture fixation. Martinez et al. [24] found that quality of life and functional outcome were significantly better after volar plate fixation compared with non-operative treatment at the two-year follow-up. On the other hand, Bartl et al. [25] found fixation led to better wrist mobility at three months, but there was no clinical difference between the treatment and the conservative group at 12 months. Similarly, Arora et al. [26] demonstrated that patients who had an operation showed better wrist function in the early postoperative period, but no significant difference was seen between the groups after.

However, the results suggest that operative fixation of distal radius fractures is better in the early stages. We feel that in patients who had a hip fracture fixation, the benefits will be much higher in terms of early mobilisation, pain comfort, functional independence, and prevention of complications. Patients would have the possibility of undergoing one anaesthetic to fix both lower and upper limb fractures, but this would depend on several factors, including ASA score and fitness for surgery. All but one patient was managed conservatively in terms of their upper limb fracture, and for this study, this patient who required fixation was excluded. Examining patients with conservative management, we found that it made little difference when looking at their KPIs compared to the University of Wales Hospital average, as well as to the NHFD average. Our patients scored higher than the NHFD average in three of the categories analysed, including prompt orthogeriatric review (92% vs. NHFD 85%), not delirious postoperatively (87% vs. NHFD 58%), and return to the original residence (79% vs. NHFD 71%). A study by Mosk et al. [27] showed that functional dependency has been a significant risk factor for patients who suffer from delirium postoperatively.

Rehabilitation following fractures is crucial in preventing postoperative complications, achieving independence of activities of daily living, and optimising functional recovery [28]. Functional autonomy and recovery of independence after sustaining fractures are crucial outcomes from the patient’s perspective. Hip surgery takes a significant toll on the body, and it is difficult for elderly patients to start mobilising postoperatively after a hip fracture. Having an associated upper limb fracture makes this task a lot more difficult. This can then have a further impact on a patient by leading to complications in their stay, such as hospital-acquired pneumonia, deep vein thrombosis or pulmonary emboli due to decreased mobility, or even death in some cases. In the study, three patients died while in hospital following their operation, showing a similar 30-day mortality rate compared to the national average.

Neck of femur fractures are associated with significant morbidity, mortality, loss of independence, and financial burden as it is expected to delay rehabilitation for walking if both fractures occur at the same time. With pressures being put on hospital beds and the rising cost of stays, it was found that the average cost of a National Health Service (NHS) bed is 300-400£ per night, when all factors are considered [22]. The NHFD Annual Report 2017 reports a mean acute ward length of stay of 16.6 days for all hip fracture patients, a similar figure to our 18.8 days for isolated hip fractures. With the length of stay almost tripled (from 16.6 to 44.4 days), one can see this has a very big effect on costs in the NHS system. It raises the argument if these upper limb fractures should be fixed to help speed up mobilisation and recovery, and ultimately reduce the financial burden on the NHS for long admissions of patients.

The main limitation of our study was that the data were collected from one centre. Although this was a tertiary centre, it would be valuable to look at data over entire health boards and compare these figures to the national average. In our study, as outlined previously, the limited number of studies on concurrent neck of femur and upper limb fractures could be seen as another limitation. Furthermore, there are limited studies showing whether it is beneficial to fix an upper limb fracture, with an associated neck of femur fracture, in the elderly population. It is difficult to say whether length of stay would have decreased in our study if this had been the case. There have also been no previous studies which have analysed the KPIs in concurrent neck of femur and upper limb fractures and compared them to a standard neck of femur population.

## Conclusions

The results demonstrate that there is a significant increase in the length of stay in the hospital for patients who sustain both a neck of femur fracture and an associated upper limb fracture. It demonstrates a positive correlation between upper limb fractures having a direct impact on the progress of physiotherapy and a delay in mobilization. On the contrary, some studies have found that concomitant upper limb fractures did not influence the length of hospitalization, in-hospital mortality, complication rate, and function. However, hip fractures and comorbidities predicted the incidence of complications and patients’ function. Surgeons should be aware that these subgroups of patients may be more likely to require rehabilitation and will also likely require signiﬁcantly longer rehabilitation duration than patients with an isolated hip fracture.
